# Environmental Risk Factors for Bipolar Disorders and High-Risk States in Adolescence: A Systematic Review

**DOI:** 10.3390/medicina56120689

**Published:** 2020-12-11

**Authors:** Giulia Menculini, Pierfrancesco Maria Balducci, Luigi Attademo, Francesco Bernardini, Patrizia Moretti, Alfonso Tortorella

**Affiliations:** 1 Department of Psychiatry, University of Perugia, Piazzale Lucio Severi 1, 06132 Perugia, Italy; giulia.menculini@libero.it (G.M.); balducci.pierfrancesco@gmail.com (P.M.B.); patrizia.moretti@unipg.it (P.M.); 2 CSM Terni, Department of Mental Health, AUSL Umbria 2, Via Bramante 40, 05100 Terni, Italy; 3 SPDC Potenza, Department of Mental Health, ASP Basilicata, Italian National Health Service, Via Petrone, 85100 Potenza, Italy; luigi.attademo@hotmail.it; 4 Department of Mental Health, AsFO Friuli Occidentale, Via Vecchia Ceramica 1, 33170 Pordenone, Italy; francescobernardini78@yahoo.fr

**Keywords:** bipolar disorder, adolescence, youth, early-onset bipolar disorder, high-risk states, environment, risk factors

## Abstract

*Background and objectives:* A deeper comprehension of the role that environmental risk factors play in the development of adolescent Bipolar Disorder (BD), as well as in the evolution of high-risk states for BD, may entangle further prevention and treatment advances. The present systematic review is aimed at critically summarizing evidence about the role that environmental risk factors play in the development of BD in adolescence and their interaction with BD high-risk states. *Materials and Methods:* MEDLINE/Pubmed, Scopus and Web of Science datasets were systematically searched until 4 September 2020. Original studies that reported information about the role of environmental risk factors in the development of BD during adolescence, or assessing their influence on the development of psychopathology in high-risk states for BD, were considered for inclusion. Two blind researchers performed title/abstract, full-text screening, and hand-screening of relevant references. The risk of bias was assessed by means of the Newcastle-Ottawa Scale. *Results:* Fourteen studies were included in the review. Negative stressful life events, particularly sexual and physical abuse, but also emotional mistreatment, were associated with more severe psychopathology in adolescents with BD, as well as with higher risk for developing mood disorders in BD offspring. Similar findings were detected for familial environment-related features, such as parental rejection and low perceived care, while no univocal results were found when analyzing familial functioning. *Conclusions:* The present systematic review confirmed the relevant role that environmental risk factors, particularly negative stressful live events and family-related features, play in the development of BD psychopathology during adolescence. Future studies are expected to clarify possible further environmental factors that may be implicated in the development of BD during youth that may serve as target of prevention and early treatment strategies.

## 1. Introduction

Bipolar disorder (BD) represents a serious mental illness affecting the global functioning of subjects at different ages. The prevalence of early-onset BD, defined by the onset occurring before the age of 18, significantly increased during the last years, despite being considered a rare clinical entity in the past [1]. The presentation of BD in youth populations is characterized by mood episodes and increased irritability, also being responsible for a significant functional impairment, as well as for a higher risk for developing adult BD. Hence, early-onset BD represents a severe clinical phenotype accounting for high suicide rates, comorbidity with anxiety and psychosis, substance abuse, and rapid cycling [2]. The correct identification of BD during childhood and adolescence represents a crucial issue [3], not only due to the fact that the first presentations of the disorder occur before the age of 18 in 70% of the cases [4], but also considering that early trajectories of the illness may be non-specific, and may thus, result in a diagnostic delay up to 10 years [5]. In addition, the missed diagnosis of BD during adolescence and young adulthood jeopardizes the possibility for targeting adequate treatments during a crucial time window [6]. Furthermore, the understanding of potential determinants of risk in early development of BD enhances therapeutic aspects, and may also help implementing preventive interventions [7].

The development of BD is strongly influenced by genetic factors, as demonstrated by twin and family studies underlining the presence of an inheritable component up to 58% [8,9]. Nonetheless, complex interactions between genes and environment may significantly contribute to the etiology of BD, possibly regulating epigenetic mechanisms [10]. Previous literature considered the role of environmental determinants in the development of BD, with preliminary evidence concerning a heterogeneous range of risk factors that encompass perinatal infections, childhood trauma and adversity, physical comorbidities, and urbanization-related factors [11,12,13,14,15,16]. Noteworthy, the exposure to traumatic events during childhood appeared to affect specific brain regions in subjects affected by BD, as demonstrated by Magnetic Resonance Imaging (MRI) studies where hippocampal and amigdalar regions resulted to be altered in BD patients exposed to childhood trauma [17,18]. Despite this, the meaning of environmental triggers is still considered to be unspecific and no univocal results hinder the inclusion of the above-mentioned risk factors as definitive markers of the illness [19]. To the best of our knowledge, no systematic review focused on the influence that putative environmental risk factors may play in the development of BD in adolescence. Previous reports considered possible environmental determinants that may increase the risk for developing BD in the general population, without providing a specific spotlight on young subjects [20,21]. Moreover, there is still a lack of evidence about the role that the environment plays in the trajectories of illness of high-risk states for BD.

The better identification of potentially modifiable risk factors for the development of BD in adolescence represents a crucial issue in the context of a better understanding of the disorder, also due to its severe clinical presentation and to its implications in terms of illness course and overall burden. In addition, a deeper comprehension of the role that environmental risk factors play in the evolution of high-risk states for BD may entangle prevention and treatment advances. Therefore, the aim of the present systematic review is to critically summarize the evidence about the role of environmental risk factors in the development of BD during adolescence and their interaction with high-risk states for BD.

## 2. Materials and Methods

The present review was conducted according to the Preferred Reporting Items for Systematic Reviews and Meta-Analyses (PRISMA) statement [22].

### 2.1. Literature Search

A systematic search of the electronic databases MEDLINE/PubMed, Scopus and Web of Science was conducted from inception to 4 September 2020, using the following search string: ((((bipolar disorder [MeSH Terms]) AND (bipolar disorder)) AND (adolescen*)) AND (environment)) AND (risk factor*).

Two blind investigators (GM and PMB) performed the literature search, title/abstract screening, full-text review. The obtained references were cross-checked and the reference list of selected articles was screened in order to search for additional literature. Discrepancies were resolved through consensus. A third investigator was consulted (AT) whenever a final decision could not be made.

### 2.2. Study Selection

Observational studies investigating the role of environmental risk factors in the development of BD during adolescence were included in the present review. In addition, we included papers reporting information about the influence of the environment on specific aspects of BD in adolescence. Reports assessing the interplay between environmental risk factors and the development of psychopathology in high-risk states for BD, namely first-degree relatives of subjects affected by BD, were also included. Studies considering parents affected by non-BD mood disorders were not included, unless they served as control group. No language restriction was applied. Grey literature was included if sufficient information was provided. Articles presenting only an opinion or hypothesis without empirical investigation, retrospective studies, reviews, letters to the editor, and case reports were excluded. Studies reporting data about genetic risk factors and peripheral biomarkers were not deemed eligible for the present review, as well as interventional studies and research focusing on BD as a possible risk factor for other disorders.

Whenever different papers referring to overlapping samples were analyzed, more than one paper was considered for inclusion if relevant information was provided for the aims of the present review. Reports investigating the development of psychiatric disorders among high-risk adolescent populations were included only if reporting information about the development of mood disorders, particularly BD, or psychopathological features possibly connected to the development of BD.

### 2.3. Data Extraction

Two blind researchers performed data extraction (LA and FB). In order to address the objectives of the review, the following information was extracted from the included papers: First author name, year and country of publication, analyzed period of time, study sample, study methodology, analyzed risk factors, and results.

### 2.4. Risk of Bias Assessment

The quality of the evidence provided by eligible studies was assessed by the two independent researchers (GM and PMB). The Newcastle-Ottawa Scale was employed for risk of bias assessment in cohort studies, considering the selection of the study groups, the comparability and the outcome assessment [23]. As for cross-sectional studies, a readapted version of the scale was adopted [24].

## 3. Results

### 3.1. Systematich Search Results

**Figure 1:**
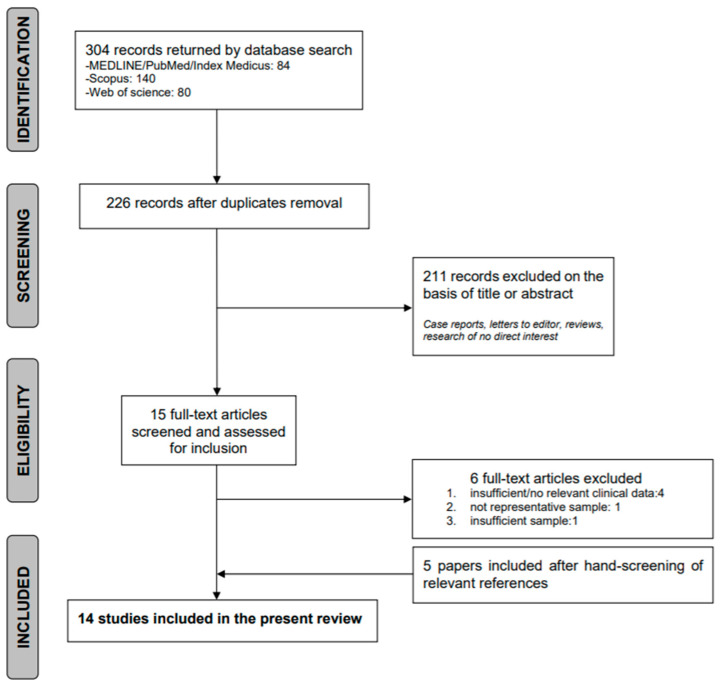
PRISMA flow-diagram.

The search initially yielded 304 records. Among these, 78 were identified as duplicates and were subsequently excluded. After performing the title and abstract screening, 15 papers were deemed eligible for further evaluation. The full text examination led to the final selection of 9 papers. The hand search of references led to the inclusion of 5 further records. The final selection led to the inclusion of 14 papers in the present review (see flowchart in Figure 1).

### 3.2. Content Results

Results are presented according to the analyzed population, with different sections for adolescents diagnosed with BD, that were considered by four of the selected studies, and offspring of subjects with BD, both affected and non-affected by psychiatric disorders, evaluated in eleven papers. Data extracted from the selected studies is summarized in Table 1 and Table 2.

#### 3.2.1. Environmental Risk Factors in Adolescents with BD

In a large sample of youths aged 7–17 the total number of negative life events was detected to be significantly higher in BD subjects than healthy controls when performing bivariate analyses (mean number of negative life events 5.5 ± 0.3 vs. 2.3 ± 0.2). Although, no significant differences were detected when comparing BD youths to those affected by depression and anxiety. In this population, psychiatric comorbidities, namely conduct disorders, anxiety disorders, Attention Deficit and Hyperactivity Disorder (ADHD), and Oppositive Defiant Disorder (ODD), were associated with the total number of negative live events at the binomial regression model [25]. In the same sample, the rates of physical and sexual abuse were specifically analyzed, presenting a prevalence of 20.6% and a significant association with Post-Traumatic Stress Disorder (PTSD) (Odds Ratio (OR) 8.8, Confidence Interval (CI) 95% 3.1–25.1), non-intact family (OR 2.6, CI 95% 1.4–5), conduct disorder (OR 2.3, CI 95% 1.1–4.8), psychotic symptoms (OR 2.1, CI 95% 1.1–3.6), longer duration of illness (OR 1.12, CI 95% 1.03–1.2) [26]. Several variables, accounting for health-, family-, and social-related factors, predicted a lower functioning in a population of adolescents aged 15–18 with a BD diagnosis [27]. Particularly, the presence of comorbidities, poor premorbid social relationships, low religious activity, and risky sexual behaviors, as well as the lack of a good relationship with siblings, determined lower scores at the Children’s Global Assessment Scale (C-GAS) [28]. 

One of the selected studies investigated the association between suicidal ideation and family environment in a sample of BD youths, where the prevalence of suicidal ideation resulted to be 36%. The study dedicated particular attention to familial cohesion (the level of warmth and intimacy between family members), adaptability (the ability of the family to change in response to stressful situations), conflicts, familial stressful events and familial psychiatric history [29]. Current suicidal ideation, demonstrated to be more frequently connected to maternal conflicts, scarce adaptability of the familial context, as measured by the Family Adaptability and Cohesion Evaluation Scale–II (FACES-II) [30], and stressful familial events during the year prior to evaluation. While, familial psychiatric history did not distinguish subjects who presented suicidal ideations from those who did not.

#### 3.2.2. Environmental Risk Factors in BD High-Risk Adolescents

In a population of BD offspring considered as genetically high-risk subjects, family environment was analyzed as a possible risk factor influencing illness trajectories, in absence of significant differences in family cohesion, parental care, and parental overprotection among the high-risk subjects and controls [31]. In the same study, subjects in the BD offspring group appeared to present with significantly higher internalizing and externalizing problems, as reported by both offspring and parents. Internalizing problems were associated with lower paternal and maternal care, whilst externalizing problems were only linked to lower maternal care, but the family environment did not mediate the relationship between BD high-risk status and dimensional psychopathology at the multi-level mediation analysis. In a 12-year longitudinal study, 52.9% of adolescent offspring of a parent with BD developed a mood disorder. The occurrence of mood disorders in this population displayed a significant association with childhood trauma, particularly emotional maltreatment, whilst family functioning did not seem to predict the onset of a mood disorder [7]. Moreover, in the same sample stressful life events as measured by the Life Events and Difficulties Schedule (LEDS) in its adolescent readaptation [32] were found to be strong predictors of the development of a first mood episode, also in absence of a clear-cut BD diagnosis, in BD offspring, together with a passive coping style and harm-avoidant temperament [33]. In a study considering early parent-child relationships, maternal neglect predicted the onset of a mood disorder in a BD high-risk population [34]. In the same sample, offspring emotionality evaluated as a temperamental trait was associated with the hazard of mood disorders, also being influenced by duration of exposure to parental BD. Similarly, the perception of maternal and paternal rejection was associated with psychopathology and with the development of BD in adolescents grown up in parental BD families [35]. In a study considering offspring of parents with BD, both affected and non affected by major psychiatric disorders, particularly mood disorders, the presence of lower cohesion, intellectual-cultural and active-recreational orientation, as well as higher conflict and control were demonstrated in families with affected offspring [36].

Preliminary results from a prospective high-risk cohort study outlined the development of a mood disorder during adolescence in 27% BD offspring from the analyzed population [37]. The relationship between adverse events and the emergence of mood disorders was evaluated in this study by means of a time-dependent variable, namely the life event load, indicating the exposure load from all stressful life events that occurred up to a particular point in time. Even though the life event load was calculated according to four different models, in the study sample the presence of stressful life events displayed a signification association with the onset of a mood disorder not depending on the employed model, with approximately 10% increased risk per unit life event load. Furthermore, the risk related to the negative stressful events was not influenced by familial loading. Higher risk for developing BD during the following 5 years in high-risk offspring, predicted by means of a risk calculator, was also associated with negative stressful life events as evaluated by a self-administered questionnaire, that appeared to influence emotion and reward processing in amygdala and occipital regions [38]. Stressful life events presented a higher prevalence in offspring of BD subjects when compared to healthy controls offspring (mean number of negative life events 1.50 ± 1.42 vs. 0.76 ± 0.97), but this prevalence was not significantly different than was demonstrated for offspring of individuals affected by non-BD Axis I disorders [39]. Furthermore, both frequency and severity of stressful life events were associated to the current diagnosis of an Axis I disorder, including BD, among high-risk offspring. In a study conducted among BD offspring aged 8–25, the majority developed a psychiatric disorder, with a higher prevalence for BD. In this high-risk affected group, more negative life events were reported, whilst early losses were not linked to a higher risk for developing clear-cut psychopathology [40].

### 3.3. Risk of Bias Assessment

Studies included in the present review showed a relatively low risk of bias as measured by the two versions of the Newcastle-Ottawa Scale (see Table 3 and Table 4). When assessing risk of bias for cohort studies, major methodology flaws were related to outcome assessment, particularly to the length of follow-up and adequacy of follow-up cohorts. As for cross-sectional studies, the risk of bias was mainly related to sample size, adequate description of non-responders and comparability.

## 4. Discussion

The present systematic review assessed the influence of non-genetic risk factors in the context of adolescent BD, not only focusing on populations of subjects with full-blown presentation of the disorders, but also considering the interplay between environmental and hereditary load in the possible evolutions of high-risk states for BD in adolescence. The identification of risk factors, during early stages of BD and in high-risk states presents several implications, due to the pleomorphic nature of such conditions and to the higher efficacy that the early administration of psychiatric treatments may demonstrate [3,41,42]. The influence of environmental risk factors on adolescent BD was confirmed by the analyzed studies, thus, endorsing the hypothesis of a multifactorial etiopathogenesis for BD during early life stages [43].

Negative stressful life events appeared to be strongly related to the development of mood disorders during the follow-up of BD high-risk populations [36,37,39], even when controlling for the familial component [38]. Furthermore, negative life events accounted for features of higher illness severity in adolescents with an already established diagnosis of BD, as demonstrated by higher comorbidity rates, more complex psychopathological presentation, longer illness duration, also impacting on functioning [25,26,27]. The higher risk for BD after stressful life events was already demonstrated in adult BD, especially when those events were proximal to the onset of the disorder [20,44]. This class of risk factors also deserves particular attention due to its influence on illness course, partly confirmed for adolescent populations by results from the present review. Indeed, stressful life events may also represent predictors for BD relapses and point at longer time until recovery [45]. At the same time, stressful life events may also be a consequence of mood episodes, with relevant impact on overall functioning [45]. Due to the controversial nature of this association, future prospective studies should further clarify this relationship in adolescent BD populations, also focusing on subsyndromal manifestations of the disorder. On the other side, stressful life events represented risk factors for more severe psychopathology in BD offspring also for what concerns the development of other disorders [38]. This could be due to the pluripotent nature that high-risk states themselves may display, with different disorders that could crystallize overtime and with the possibility for comorbid disorders to present as adjunctive outcomes [19]. Due to the scarce specificity that these putative risk factors display when analyzed within an entire group, future studies should probably improve individual risk assessment, e.g., by means of optimized risk calculators, in the attempt to advance personalized intervention strategies [46,47]. The role of environmental risk factors in a precision psychiatry framework is validated by the demonstrated interplay between negative events, risk of developing BD and functional alterations in specific neuroanatomic regions, such as hippocampus, amygdala and frontoparietal areas [37]. Such a relationship may be illustrative of the complex interactions underpinned by the emergence of BD during youth, that could be better understood by means of the identification of neuroimaging and peripheral biomarkers [48,49,50,51]. The combination of these biological factors, together with familial and environmental markers should be considered in the future in order to define individual risk, identifying a “risk syndrome” that could increase the likelihood of the progression to BD [52].

Due to the already-mentioned overall high severity of BD when presenting during early stages of life [2], the identification of modifiable environmental factors represent a crucial issue, in order to target prevention strategies. For this purpose, the recent efforts that some countries made to denounce childhood physical and sexual abuse by strengthening their public policies on the topic may be interpreted as the first step towards larger campaigns aimed at preventing psychiatric disorders among youth populations [53,54]. In the context of childhood traumatic events, physical as well as emotional abuse should be considered as strong determinants of BD development, as demonstrated by the results from some of the included reports regarding parental rejection, neglect, and low care [7,30,33,34]. The role of emotional abuse was demonstrated in adult BD samples as the preferential trauma subtype associated with later development of the disorder, probably influencing emotional regulation and stress reactivity through changes in catecholaminergic responses [55]. A better stratification of childhood trauma and a standardization of the assessment instruments will hopefully further clarify this association also in adolescent and high-risk populations.

Further studies focusing on the role of traumatic experiences in the development of BD may also present implications in consideration of the frequent comorbidity between BD and PTSD. In fact, the two disorders were also demonstrated to share common genetic heritage that two may be mediated by a variant of the Brain-Derived Neurotrophic Factor (BDNF), with possible epigenetic mechanisms that could act as response to traumatic and stressful life events [56]. Similarly, in neuroimaging studies alterations of the prefrontal cortex were detected in both conditions [57,58]. As a consequence, a better comprehension of the role that traumatic experiences play as possible mediators of BD development could also help shedding light on pathogenetic pathways that lead to the development of a clear-cut PTSD in BD subjects.

The role of family functioning was controversial according to reports included in the present review, influencing specific features of BD in adolescence, e.g., suicidal ideation, rather than the development of the disorder [29]. This finding partially contrasts with previous literature on BD [59], but more specific factors related to the familial environment, e.g., parental bonding, should be analyzed in order to elucidate the exact impact of family-related factors in BD development [60]. The better identification of the above-mentioned risk factors may also help targeting psychosocial interventions during early stages of adolescent BD, such as family-focused treatment and family-oriented psychoeducation, that already obtained positive evidence in previous literature [3,61,62]. 

Surprisingly, only few risk factors were considered in the included studies. Although medical comorbidities may play a less significant role in younger populations, the shared pathophysiological pathways leading to both mental and physical illnesses and the decrease of life expectancy caused by the medical burden in BD subjects claim further research also in youth samples [63,64]. A growing body of research is also evidence of the role of urbanization-related factors, particularly air pollution, in the development of major psychiatric disorders [11], as well as their influence on mental illnesses relapses [65]. Consequently, further research should better clarify the relationship between urbanicity and air pollution and adolescent BD. 

To our best knowledge, the present review presents the first effort to systematically summarize the evidence about environmental risk factors in youths affected by BD and high-risk populations, in the attempt to enhance knowledge in this still under-studied area of investigation. Potential strengths of this report include the use of a systematic methodology following PRISMA guidelines, the inclusion of studies considering not only full-blown BD, but also high-risk populations, and the methodological validity of most of the assessed studies. Despite this, the present review presents limitations. First, the different study designs and the heterogenous populations in terms of age limited the possibility of inter-study comparisons. Small sample sizes in some of the included studies represented a further limitation to the generalizability of findings. As for outcome assessment, we did not select studies on the basis of specific instruments, e.g., semi-structured interviews, thus including reports evaluating symptoms by means of auto-administered tools that may increase subjectivity of findings. Furthermore, the indirect exclusion of subjects that may present with unipolar depressive disorders during adolescence did not allow crucial issues in adolescent BD to be assessed, such as antidepressant use and the unipolar-bipolar conversion.

## 5. Conclusions

The present systematic review confirmed the relevant role that environmental risk factors play in the development of BD psychopathology, with particular attention to adolescent and high-risk populations. Although the function of negative stressful life events and family-related factors appeared to be crucial, future studies should hopefully better stratify risks related to the above-mentioned determinants, as well as possible further environmental factors that may be implicated in the development of BD during youth. The better identification of the complex pathogenetic pathways that lead to full-blown manifestations of BD may help targeting preventing strategies and thus contribute to the modification of unfavorable illness outcomes.

## Figures and Tables

**Table 1:** Selected studies concerning influence of environmental risk factors on Bipolar Disorders in adolescence.

References	Country	Study Design	Sample	Assessment Instruments	Considered Environmental Factors	Results
Goldstein et al., 2009	USA	4-year longitudinal study	446 outpatients and inpatients (7–17) with BD-I, BD-II or BD-NOS according to DSM-IV (Course and Outcome of Bipolar Illness in Youth study)	DSM-IV K-SADS-P K-SADS-PL K-SADS-MRS CBQ FACES-II LEC FHS GIS C-GAS Clinical interview	SES Living with both parents History of physical and sexual abuse Family conflict Family closeness Family adaptability Family stress	BD youth with current suicidal ideation (*n* = 160, 36%) reported more conflict with their mother (mean CBQ score 4.9 ± 5.2 vs. 6.0 ± 5.4, *p* = 0.04) and less family adaptability (mean FACES-II score 44.3 ± 9.1 vs. 42.4 ± 9.2). A significantly higher rates of stressful family events was also reported, in particular: illness of a family member (40% vs. 28%, *p* = 0.04), death of a family member 35% vs. 24%, *p* = 0.03), increased absence of a parent from home (27% vs. 14%, *p* < 0.01), and trouble with a sibling (51% vs. 39%; *p* = 0.03).
Romero et al., 2009a	USA	Cross-sectional study with data from a 6-year period	446 outpatients and inpatients (7–17) with BD-I, BD-II or BD-NOS according to DSM-IV (Course and Outcome of Bipolar Illness in Youth study) 65 youths with depression/anxiety disorders (DEP/ANX group) 65 HCs youths	K-SADS-PL LEC	Negative dependent, independent, and uncertain life events	Subjects with BD reported a similar rate of NLEs as DEP/ANX group (mean number 5.5 ± 0.3 vs. 6.1 ± 0.5, NS). Both groups had more NLEs than HCs (mean number 5.5 ± 0.3 vs. 2.3 ± 0.2, *p* < 0.001). NLEs were associated with lower socioeconomic status (*p* = 0.005), non-intact family (*p* = 0.003), and psychiatric comorbidity, namely conduct disorders (*p* = 0.003), anxiety disorders (*p* = 0.02), ADHD (*p* = 0.03), and ODD (*p* = 0.03).
Romero et al., 2009b	USA	Cross-sectional study	446 outpatients and inpatients (7–17) with BD-I, BD-II or BD-NOS according to DSM-IV (Course and Outcome of Bipolar Illness in Youth study)	K-SADS-PL, PTSD section FHS Hollingshead four-factor scale	SES Intact family History of physical and sexual abuse	Sexual and/or physical abuse were found to be common (*n* = 92, 20.6%; physical abuse: *n* = 40, 9%, sexual abuse: n = 30, 7%, both: *n* = 22, 5%). Physical abuse was associated with PTSD (OR 10.4, CI 3.2–34.4), non-intact family (OR 4.2, CI 1.5–11.7), first-degree family history of mood disorder (OR 3.4, CI 1.1–10.6), and psychosis (OR 2.3, CI 1.1–5). Sexual abuse was associated with PTSD (OR 7.8, CI 2–30.4). Subjects with both types of abuse were older (*p* = 0.015), with longer illness duration (*p* = 0.01), non-intact family (*p* = 0.003), and greater prevalence of PTSD (<0.001) and CD (*p* = 0.03) as compared with the non-abused group.
Bakare et al., 2011	Nigeria	One-year cross-sectional study	46 outpatient adolescents (15–18) with BD-I or BD-II diagnosed according to DSM-IV	Socio-demographic questionnaire C-GAS Clinical interview	Marital status of the parents Premorbid peer relationship Religion activities History of sexual risky behavior Relationship with siblings Substance use	Poor premorbid peer relationship (*p* < 0.001), poor relationship with siblings (*p* < 0.001), low level of religion activities (*p* < 0.001), and history of sexual risky behavior (*p* < 0.001), comorbidities (*p* < 0.001), and number of hospital admissions (*p* < 0.001) were significantly associated with lower functioning.

Notes: ADHD = Attention Deficit/Hyperactivity Disorder; BD = Bipolar Disorder; CBQ = Conflict Behavior Questionnaire; CD = Conduct Disorder; C-GAS = Children’s Global Assessment Scale; CI = Confidence Interval; FHS = Family History Screen; FACES-II = Family Adaptability and Cohesion Evaluation Scale-II; GIS = General Information Sheet; HCs = Healthy Controls; HEIC = Home Environment Interview for Children; K-SADS-BP = Kiddie Schedule for Affective Disorders and Schizophrenia, Bipolar Disorder Version; K-SADS-MRS = Kiddie Schedule for Affective Disorders and Schizophrenia, Mania Rating Scale; K-SADS-P = Kiddie Schedule for Affective Disorders and Schizophrenia, Present Version; K-SADS-PL = Kiddie Schedule for Affective Disorders and Schizophrenia, Present and Lifetime Version; LEC = Life Events Checklist; NLEs = Negative Life Events; NS = Not Significant; ODD = Oppositive-Defiant Disorder; OR = Odds Ratio; PTSD = Post Traumatic Stress Disorder; SES = Socioeconomic Status.

**Table 2:** Selected studies concerning influence of environmental risk factors on the development of psychopathology in adolescents at high-risk for Bipolar Disorder.

References	Country	Study Design	Sample	Assessment Instruments	Considered Environmental Factors	Results
Hillegers et al., 2004	The Netherlands	Cohort study (preliminary findings from a first 16-month assessment)	140 adolescents (12–21), offspring of 86 BD parents (Dutch Bipolar Offspring Study)	DSM-IV K-LEDS FH-RDC Life event load (time-dependent variable calculated according to four different models)	Stressful life events	27% of youth included in the sample developed a mood disorder during follow-up (median age 14). The life event load, not depending on the model, was associated with a 10% increased risk for mood disorders (HR = 1.1), with no modification of the significant relationship after controlling for the familial component.
Duffy et al., 2006	Canada	Cross-sectional study	126 (8–25) youths, offspring of BD subjects. Parents were stratified according to lithium response (responders = 36, non-responders = 27)	K-SADS-PL Semi-structured interview by Goodyear et al. for life events, difficulties, and permanent losses EAS	Stressful life events Early permanent losses	The number of NLEs was higher in affected BD offspring when compared to unaffected ones (mean number of NLEs 1.50 ± 1.42 vs. 0.76 ± 0.97, *p* = 0.03). This result was not replicated for early losses. This relationship was detected to be mediated by emotionality, which contributed to lifetime mood disorders at the stepwise regression analysis (OR 2.47, 95% CI 4.56–1.34, *p* < 0.01).
Reichart et al., 2007	The Netherlands	Cross-sectional study (third, 5-year measurement of a longitudinal prospective study)	129 offspring of 80 BD parents (Dutch Bipolar Offspring Study) 1122 young adults from the general population	DSM-IV K-SADS-PL EMBU Clinical history features for BD course characteristics	Parental rearing behavior Parental psychopathology	Offspring with a father affected by BD perceived more rejection than those with a mother with BD (mean scores at the EMBU Rejection subscale: 9.33 vs. 8.19, *p* < 0.05). The development of psychopathology in BD offspring was associated with higher rates of parent rejecting (unstandardized coefficient B = 2.82 for father, B = 1.29 for mother, *p* = 0.05).
Ferreira et al., 2013	Brazil	Cross-sectional study	46 subjects (18–65) diagnosed with BD-I according to DSM-IV and their offspring (6–17) 30 healthy subjects (18–65) and their offspring (6–17)	K-SADS-PL FES	Family environment	BD families were characterized by lower cohesion (*p* = 0.001), intellectual-cultural orientation (*p* = 0.005), active-recreational orientation (*p* = 0.002), moral-religious emphasis (*p* = 0.004) and organization (*p* < 0.001), and higher conflict (*p* < 0.001). Offspring of BD parents presented more frequent development of Axis I disorders (BD = 12.8%). In the affected group, lower cohesion (*p* = 0.003), intellectual-cultural orientation (*p* = 0.01) and active-recreational orientation (*p* = 0.007) and higher conflict (*p* = 0.001) and control (*p* = 0.01) were detected.
Kemner et al., 2015	The Netherlands	Cohort study with a 12-year follow-up	140 adolescents (12–21), offspring of 86 BD parents (Dutch Bipolar Offspring Study)	DSM-IV K-SADS-PL LEDS TCI UCL Short-EMBU	Stressful life events	24% BD offspring developed BD, with a 54% incidence of mood disorders during follow-up. First mood episode was associated to the total load of stressful life events (HR = 1.14). Subsequent episodes, although to a lesser extent, were as well associated with life events (HR = 1.12). Passive coping style increased the risk of mood episode onset and recurrent episodes HR = 1.23, HR = 1.18), but also altered the effect of life events on mood disorders.
Doucette et al., 2016	Canada	Cohort study with a 25-year follow-up	233 offspring (mean age 16.6) of BD parents	DSM-IV K-SADS-PL AMI CECA.Q EAS LEQ Hollingsead SES Scale Self-report measures of temperament and early adversities	Early childhood adversity Emotionality Exposure to parental BD Stressful life events	Perceived maternal neglect predicted mood and anxiety disorders onset in high-risk adolescents (HR = 1.1, 95% CI 1.0–1.2), even after adjusting for further factors, e.g., exposure to parental BD. High offspring emotionality appeared to be as well associated to the development of mood disorders (HR = 1.7, 95% CI 1.9–3.1), also being the possible mediator of the relationship between maternal neglect and the development of psychopathology.
Pan et al., 2017	USA	Cross-sectional study of a sample from a 7-year cohort study	Offspring (13–18) of parents affected by BD (*n* = 269), non-BD Axis I disorders (*n* = 88), and offspring of HCs (n = 81) (Pittsburgh Bipolar Offspring Study)	DSM-IV K-SADS-PL SLES	Stressful live events during the year before evaluation	Offspring of BD probands had greater number of severe stressful life events than HCs offspring (mean (SE) 13.89 (0.05) vs. 10.22 (0.08), *p* = 0.001), but not non-BD offspring. Total number and severity of negative stressful life events was associated with higher rates of Axis I disorders in both BD and non-BD affected probands.
Lau et al., 2018	Australia	Cross-sectional study of a sample from a cohort study	146 offspring (12–21): High-risk (*n* = 90) BD offspring and control (*n* = 56) offspring (Bipolar Kids and Sibs Study)	DSM-IV-TR K-SADS-BP FIGS DIGS v.4 FACES-II PBI CBCL/ 6—18 YSR ABCL/18–59 ASR	Family cohesion Parental bonding	BD offspring presented significantly higher internalizing and externalizing problems, both on self- (Int: M (SE) 55.95 (1.31) vs. 48.27 (1.74), *p* = 0.001, Ext: M (SE) 53.74 (1.19) vs. 49.00 (1.52), *p* = 0.03) self- and parent- (Ext: 56.37 (4.71) vs. 47.17 (6.36), *p* = 0.029) reports. Internalizing problems displayed a significant association with low maternal (*p* = 0.025) and paternal (*p* = 0.025) care, whilst externalizing problems were associated with low maternal care (*p* = 0.011). Family environment-related factors did not mediate the relationship between high-risk BD state and dimensional psychopathology.
Hanford et al., 2019	USA	Cross-sectional study of samples from larger cohort studies	22 offspring (mean age 14.1 ± 2.4 years) of BD parents, 22 offspring of healthy controls (mean age 13.7 ± 1.8 years) (Pittsburgh Bipolar Offspring Study, Longitudinal Assessment of Manic Symptoms)	DSM-IV K-SADS-PL K-SADS-MRS CALS SCARED C-GAS SLES Predictive 5-year risk calculator MRI for investigation of emotional and reward networks	Stressful live events	Higher risk calculator score showed greater positive associations between number of recent exposure to negative stressful life events and activity within bilateral fusiform gyri (right: Z = 5, *p* < 0.001, left: Z = 3.6, *p* < 0.001) and the right amygdala (Z = 4.1, *p* < 0.001). Risk calculator score alone showed positive relationships with activity in bilateral lateral occipital cortices (right: Z = 3.7; *p* < 0.001; left: Z = 4.0 *p* = 0.001).
Koenders et al., 2020	The Netherlands	12-year cohort study	102 offspring (12–21) of parents with BD (Dutch Bipolar Offspring Study)	DSM-IV K-SADS-PL QFP CTQ	Childhood trauma Family functioning	52.9% (*n* = 54) offspring developed a mood disorder, of which 12.7% (*n* = 24) developed BD. Emotional maltreatment was significantly associated with mood disorder onset (HR = 1.07, 95% CI 1.18–2.82, *p* = 0.007). Family functioning, nor its subscales, shows significant associations with mood disorder onset (HR = 1.01, 95% CI: 0.89–1.14, *p* = 0.889.

Notes: ABCL/18–59 = Adult Behavior Checklist for Ages 18–59; AMI = Affective Morbidity Index; ASR = Adult Self-Report; BD = Bipolar Disorder; CALS = Children Affective Liability Scale; CBCL/6–18 = Child Behavior Checklist for Ages 6–18; CBQ = Conflict Behavior Questionnaire; CD = Conduct Disorder; CECA.Q = Childhood Experience of Care and Abuse Questionnaire; C-GAS = Children’s Global Assessment Scale; CI = Confidence Interval; CTQ = Child Trauma Questionnaire; DIGS = Diagnostic Interview for Genetic Studies; EAS = Early Adolescence Temperament Scale; EMBU = Swedish acronym for “my memories of upbringing”; FACES-II = Family Adhesion and Cohesion Evaluation Scales-II; FES = Family Environment Scale; FH-RDC = Family History Related Research Criteria; FHS = Family History Screen; FIGS = Family Interview for Genetic Studies; HCs = Healthy Controls; HEIC = Home Environment Interview for Children; HR = Hazard Ratio; K-LEDS = Kiddie Life Events and Difficulty Scale; K-SADS-BP = Kiddie Schedule for Affective Disorders and Schizophrenia, Bipolar Disorder Version; K-SADS-MRS = Kiddie Schedule for Affective Disorders and Schizophrenia, Mania Rating Scale; K-SADS-P = Kiddie Schedule for Affective Disorders and Schizophrenia, Present Version; K-SADS-PL = Kiddie Schedule for Affective Disorders and Schizophrenia, Present and Lifetime Version; LEC = Life Events Checklist; LEDS = Life Events and Difficulties Scale; LEQ = Life Events and Difficulties Questionnaire; M = Median; PBI = Parental Bonding Instrument; QFP = Questionnaire for Family Problems; SCARED = Screening for Child Anxiety Related Disorders; SE = Standard Error; TCI = Temperament and Character Inventory; UCL = Utrecht Coping List; YSR = Youth Self-Report.

**Table 3:** Newcastle-Ottawa Quality Scale assessment for cohort studies. Stars (*) are assigned to each item if the requirement is satisfied.

Author(s) (Year)	Selection				Comparability		Outcome			Total Stars
	S1	S2	S3	S4	C1a	C1b	O1	O2	O3	
Hillegers et al., (2004)		*	*	*	*	*	*		*	7
Duffy et al., (2007)	*	*	*	*		*	*	*	*	8
Goldstein et al., (2009)	*	*			*	*	*			5
Kemner et al., (2015)		*	*	*	*	*	*	*	*	8
Doucette et al., (2016)	*	*		*	*		*	*		6
Koenders et al., (2020)	*	*		*	*	*	*	*		7

**Table 4:** Newcastle-Ottava Scale assessment readapted for cross-sectional studies. Stars (*) are assigned to each item if the requirement is satisfied. Stars (**) are assigned to each item if the requirement is satisfied.

Author(s) (Year)	Selection				Comparability		Outcome		Total Stars
	S1	S2	S3	S4	C1a	C1b	O1	O2	
Reichart et al., (2007)	*		*	**		*	**	*	8
Romero et al., (2009a)	*			**			*	*	5
Romero et al., (2009b)	*			**	*	*	**	*	8
Bakare et al., (2011)	*			**			**	*	6
Ferreira et al., (2013)	*			**			*	*	5
Pan et al., (2017)	*			**	*	*	**	*	8
Lau et al., (2018)	*			**	*	*	*	*	7
Hanford et al., (2019)	*			**		*	**	*	7

## References

[1] Connor D.F., Ford J.D., Pearson G.S., Scranton V.L., Dusad A. (2017). Early-Onset Bipolar Disorder: Characteristics and Outcomes in the Clinic. J. Child Adolesc. Psychopharmacol..

[2] Geoffroy P.A., Etain B., Jamain S., Bellivier F., Leboyer M. (2013). Early onset bipolar disorder: Validation from admixture analyses and biomarkers. Can. J. Psychiatry.

[3] Vieta E., Salagre E., Grande I., Carvalho A.F., Fernandes B.S., Berk M., Birmaher B., Tohen M., Suppes T. (2018). Early intervention in Bipolar disorder. Am. J. Psychiatry.

[4] Baldessarini R.J., Bolzani L., Cruz N., Jones P.B., Lai M., Lepri B., Perez J., Salvatore P., Tohen M., Tondo L. (2010). Onset-age of bipolar disorders at six international sites. J. Affect. Disord..

[5] Altamura A.C., Buoli M., Albano A., Dell’Osso B. (2010). Age at onset and latency to treatment (duration of untreated illness) in patients with mood and anxiety disorders: A naturalistic study. Int. Clin. Psychopharmacol..

[6] Drancourt N., Etain B., Lajnef M., Henry C., Raust A., Cochet B., Mathieu F., Gard S., M’Bailara K., Zanouy L. (2013). Duration of untreated bipolar disorder: Missed opportunities on the long road to optimal treatment. Acta Psychiatr. Scand..

[7] Koenders M.A., Mesman E., Giltay E.J., Elzinga B.M., Hillegers M.H.J. (2020). Traumatic experiences, family functioning, and mood disorder development in bipolar offspring. Br. J. Clin. Psychol..

[8] Kim H.-I., Lee H.-J., Cho C.-H., Kang S.-G., Yoon H.-K., Park Y.-M., Lee S.-H., Moon J.-H., Song H.-M., Lee E. (2015). Association of CLOCK, ARNTL, and NPAS2 gene polymorphisms and seasonal variations in mood and behavior. Chronobiol. Int..

[9] Craddock N., Sklar P. (2013). Genetics of bipolar disorder. Lancet.

[10] Uher R. (2014). Gene-environment interactions in severe mental illness. Front. Psychiatry.

[11] Khan A., Plana-Ripoll O., Antonsen S., Brandt J., Geels C., Landecker H., Sullivan P.F., Pedersen C.B., Rzhetsky A. (2019). Environmental pollution is associated with increased risk of psychiatric disorders in the US and Denmark. PLoS Biol..

[12] Marrie R.A., Reingold S., Cohen J., Stuve O., Trojano M., Sorensen P.S., Cutter G., Reider N. (2015). The incidence and prevalence of psychiatric disorders in multiple sclerosis: A systematic review. Mult. Scler. J..

[13] Leo R.J., Singh J. (2016). Migraine headache and bipolar disorder comorbidity: A systematic review of the literature and clinical implications. Scand. J. Pain.

[14] Šprah L., Dernovšek M.Z., Wahlbeck K., Haaramo P. (2017). Psychiatric readmissions and their association with physical comorbidity: A systematic literature review. BMC Psychiatry.

[15] Agnew-Blais J., Danese A. (2016). Childhood maltreatment and unfavourable clinical outcomes in bipolar disorder: A systematic review and meta-analysis. Lancet Psychiatry.

[16] Sutterland A.L., Fond G., Kuin A., Koeter M.W.J., Lutter R., van Gool T., Yolken R., Szoke A., Leboyer M., De Haan L. (2015). Beyond the association. Toxoplasma gondii in schizophrenia, bipolar disorder, and addiction: Systematic review and meta-analysis. Acta Psychiatr. Scand..

[17] Janiri D., Sani G., Rossi P., Piras F., Iorio M., Banaj N., Giuseppin G., Spinazzola E., Maggiora M., Ambrosi E. (2017). Amygdala and hippocampus volumes are differently affected by childhood trauma in patients with bipolar disorders and healthy controls. Bipolar Disord..

[18] Janiri D., Sani G., De Rossi P., Piras F., Banaj N., Ciullo V., Simonetti A., Arciniegas D.B., Spalletta G. (2019). Hippocampal subfield volumes and childhood trauma in bipolar disorders. J. Affect. Disord..

[19] Raballo A., Mechelli A., Menculini G., Tortorella A. (2019). Risk syndromes in psychiatry: A state-of-the-art overview. Arch. Psychiatry Psychother..

[20] Bortolato B., Köhler C.A., Evangelou E., León-Caballero J., Solmi M., Stubbs B., Belbasis L., Pacchiarotti I., Kessing L.V., Berk M. (2017). Systematic assessment of environmental risk factors for bipolar disorder: An umbrella review of systematic reviews and meta-analyses. Bipolar Disord..

[21] Marangoni C., Hernandez M., Faedda G.L. (2016). The role of environmental exposures as risk factors for bipolar disorder: A systematic review of longitudinal studies. J. Affect. Disord..

[22] Moher D., Liberati A., Tetzlaff J., Altman D.G., PRISMA Group (2009). Preferred Reporting Items for Systematic Reviews and Meta-Analyses: The PRISMA Statement. J. Clin. Epidemiol..

[23] Wells G.A., Shea B., O’Connell D., Peterson J., Welch V., Losos M., Tugwell P. (2014). The Newcastle-Ottawa Scale (NOS) for Assessing the Quality of Nonrandomised Studies in Meta-Analyses. www.ohri.ca/programs/clinical_epidemiology/oxford.asp.

[24] Herzog R., Álvarez-Pasquin M.J., Díaz C., Del Barrio J.L., Estrada J.M., Gil Á. (2013). Are healthcare workers intentions to vaccinate related to their knowledge, beliefs and attitudes? A systematic review. BMC Public Health.

[25] Romero S., Birmaher B., Axelson D.A., Iosif A.M., Williamson D.E., Gill M.K., Goldstein B.I., Strober M.A., Hunt J., Goldstein T.R. (2009). Negative life events in children and adolescents with bipolar disorder. J. Clin. Psychiatry.

[26] Romero S., Birmaher B., Axelson D., Goldstein T., Goldstein B.I., Gill M.K., Iosif A.-M., Strober M.A., Hunt J., Esposito-Smythers C. (2009). Prevalence and correlates of physical and sexual abuse in children and adolescents with bipolar disorder. J. Affect. Disord..

[27] Bakare M.O., Agomoh A.O., Eaton J., Ebigbo P.O., Onwukwe J.U. (2011). Functional status and its associated factors in Nigerian adolescents with bipolar disorder. Afr. J. Psychiatry.

[28] Shaffer D., Gould M.S., Brasic J., Fisher P., Aluwahlia S., Bird H. (1983). A Children’s Global Assessment Scale (CGAS). Arch. Gen. Psychiatry.

[29] Goldstein T.R., Birmaher B., Axelson D., Goldstein B.I., Gill M.K., Esposito-Smythers C., Ryan N.D., Strober M.A., Hunt J., Keller M. (2009). Family environment and suicidal ideation among bipolar youth. Arch. Suicide Res..

[30] Olson D.H., Portner J., Bell R. (1982). FACES II: Family Adaptability and Cohesion Evaluation Scales.

[31] Lau P., Hawes D.J., Hunt C., Frankland A., Roberts G., Wright A., Costa D.S., Mitchell P.B. (2018). Family environment and psychopathology in offspring of parents with bipolar disorder. J. Affect. Disord..

[32] Monck E., Dobbs R. (1985). Measuring life events in an adolescent population: Methodological issues and related findings. Psychol. Med..

[33] Kemner S.M., Mesman E., Nolen W.A., Eijckemans M.J.C., Hillegers M.H.J. (2015). The role of life events and psychological factors in the onset of first and recurrent mood episodes in bipolar offspring: Results from the Dutch Bipolar Offspring Study. Psychol. Med..

[34] Doucette S., Levy A., Flowerdew G., Horrocks J., Grof P., Ellenbogen M., Duffy A. (2016). Early parent–child relationships and risk of mood disorder in a Canadian sample of offspring of a parent with bipolar disorder: Findings from a 16-year prospective cohort study. Early Interv. Psychiatry.

[35] Reichart C.G., Van Der Ende J., Hillegers M.H.J., Wals M., Bongers I.L., Nolen W.A., Ormel J., Verhulst F.C. (2007). Perceived parental rearing of bipolar offspring. Acta Psychiatr. Scand..

[36] Ferreira G.S., Moreira C.R.L., Kleinman A., Nader E.C.G.P., Gomes B.C., Teixeira A.M.A., Rocca C.C.A., Nicoletti M., Soares J.C., Busatto G.F. (2013). Dysfunctional family environment in affected versus unaffected offspring of parents with bipolar disorder. Aust. N. Z. J. Psychiatry.

[37] Hillegers M.H.J., Burger H., Wals M., Reichart C.G., Verhulst F.C., Nolen W.A., Ormel J. (2004). Impact of stressful life events, familial loading and their interaction on the onset of mood disorders: Study in a high-risk cohort of adolescent offspring of parents with bipolar disorder. Br. J. Psychiatry.

[38] Hanford L.C., Eckstrand K., Manelis A., Hafeman D.M., Merranko J., Ladouceur C.D., Graur S., McCaffrey A., Monk K., Bonar L.K. (2019). The impact of familial risk and early life adversity on emotion and reward processing networks in youth at-risk for bipolar disorder. PLoS ONE.

[39] Pan L.A., Goldstein T.R., Rooks B.T., Hickey M., Fan J.Y., Merranko J., Monk K., Diler R.S., Sakolsky D.J., Hafeman D. (2017). The relationship between stressful life events and Axis i diagnoses among adolescent offspring of probands with bipolar and non-bipolar psychiatric disorders and healthy controls: The Pittsburgh Bipolar Offspring Study (BIOS). J. Clin. Psychiatry.

[40] Duffy A., Alda M., Trinneer A., Demidenko N., Grof P., Goodyer I.M. (2007). Temperament, life events, and psychopathology among the offspring of bipolar parents. Eur. Child Adolesc. Psychiatry.

[41] Joyce K., Thompson A., Marwaha S. (2016). Is treatment for bipolar disorder more effective earlier in illness course? A comprehensive literature review. Int. J. Bipolar Disord..

[42] Van Meter A.R., Burke C., Youngstrom E.A., Faedda G.L., Correll C.U. (2016). The Bipolar Prodrome: Meta-Analysis of Symptom Prevalence Prior to Initial or Recurrent Mood Episodes. J. Am. Acad. Child Adolesc. Psychiatry.

[43] DelBello M.P. (2018). A Risk Calculator for Bipolar Disorder in Youth: Improving the Odds for Personalized Prevention and Early Intervention?. J. Am. Acad. Child Adolesc. Psychiatry.

[44] Tsuchiya K.J., Byrne M., Mortensen P.B. (2003). Risk factors in relation to an emergence of bipolar disorder: A systematic review. Bipolar Disord..

[45] Koenders M.A., Giltay E.J., Spijker A.T., Hoencamp E., Spinhoven P., Elzinga B.M. (2014). Stressful life events in bipolar i and II disorder: Cause or consequence of mood symptoms?. J. Affect. Disord..

[46] Birmaher B., Merranko J.A., Goldstein T.R., Gill M.K., Goldstein B.I., Hower H., Yen S., Hafeman D., Strober M., Diler R.S. (2018). A Risk Calculator to Predict the Individual Risk of Conversion From Subthreshold Bipolar Symptoms to Bipolar Disorder I or II in Youth. J. Am. Acad. Child Adolesc. Psychiatry.

[47] Hafeman D.M., Merranko J., Goldstein T.R., Axelson D., Goldstein B.I., Monk K., Hickey M.B., Sakolsky D., Diler R., Iyengar S. (2017). Assessment of a person-level risk calculator to predict new-onset bipolar spectrum disorder in youth at familial risk. JAMA Psychiatry.

[48] Mourão-Miranda J., Oliveira L., Ladouceur C.D., Marquand A., Brammer M., Birmaher B., Axelson D., Phillips M.L. (2012). Pattern recognition and functional neuroimaging help to discriminate healthy adolescents at risk for mood disorders from low risk adolescents. PLoS ONE.

[49] Sugranyes G., Solé-Padullés C., de la Serna E., Borras R., Romero S., Sanchez-Gistau V., Garcia-Rizo C., Goikolea J.M., Bargallo N., Moreno D. (2017). Cortical Morphology Characteristics of Young Offspring of Patients With Schizophrenia or Bipolar Disorder. J. Am. Acad. Child Adolesc. Psychiatry.

[50] Goodday S.M., Horrocks J., Keown-Stoneman C., Grof P., Duffy A. (2016). Repeated salivary daytime cortisol and onset of mood episodes in offspring of bipolar parents. Int. J. Bipolar Disord..

[51] Singh M.K., Jo B., Adleman N.E., Howe M., Bararpour L., Kelley R.G., Spielman D., Chang K.D. (2013). Prospective neurochemical characterization of child offspring of parents with bipolar disorder. Psychiatry Res. Neuroimaging.

[52] Geoffroy P.A., Scott J. (2017). Prodrome or risk syndrome: What’s in a name?. Int. J. Bipolar Disord..

[53] Jacka F.N., Mykletun A., Berk M. (2012). Moving towards a population health approach to the primary prevention of common mental disorders. BMC Med..

[54] Berk M., Moylan S., Jacka F.N. (2014). A Royal gift to prevention efforts. Aust. N. Z. J. Psychiatry.

[55] Etain B., Mathieu F., Henry C., Raust A., Roy I., Germain A., Leboyer M., Bellivier F. (2010). Preferential association between childhood emotional abuse and bipolar disorder. J. Trauma. Stress.

[56] Rakofsky J.J., Ressler K.J., Dunlop B.W. (2012). BDNF function as a potential mediator of bipolar disorder and post-traumatic stress disorder comorbidity. Mol. Psychiatry.

[57] Librenza-Garcia D., Suh J.S., Watts D.P., Ballester P.L., Minuzzi L., Kapczinski F., Frey B.N. (2020). Structural and Functional Brain Correlates of Neuroprogression in Bipolar Disorder. Curr. Top. Behav. Neurosci..

[58] Harnett N.G., Goodman A.M., Knight D.C. (2020). PTSD-related neuroimaging abnormalities in brain function, structure, and biochemistry. Exp. Neurol..

[59] Barron E., Sharma A., Le Couteur J., Rushton S., Close A., Kelly T., Grunze H., Ferrier I.N., Le Couteur A. (2014). Family environment of bipolar families: A UK study. J. Affect. Disord..

[60] Tole F., Kopf J., Schröter K., Palladino V.S., Jacob C.P., Reif A., Kittel-Schneider S. (2019). The role of pre-, peri-, and postnatal risk factors in bipolar disorder and adult ADHD. J. Neural. Transm..

[61] Miklowitz D.J., Schneck C.D., George E.L., Taylor D.O., Sugar C.A., Birmaher B., Kowatch R.A., DelBello M.P., Axelson D.A. (2014). Pharmacotherapy and family-focused treatment for adolescents with bipolar I and II disorders: A 2-year randomized trial. Am. J. Psychiatry.

[62] Miklowitz D.J., Chung B. (2016). Family-Focused Therapy for Bipolar Disorder: Reflections on 30 Years of Research. Fam. Process.

[63] Kessing L.V., Vradi E., McIntyre R.S., Andersen P.K. (2015). Causes of decreased life expectancy over the life span in bipolar disorder. J. Affect. Disord..

[64] Correll C.U., Solmi M., Veronese N., Bortolato B., Rosson S., Santonastaso P., Thapa-Chhetri N., Fornaro M., Gallicchio D., Collantoni E. (2017). Prevalence, incidence and mortality from cardiovascular disease in patients with pooled and specific severe mental illness: A large-scale meta-analysis of 3,211,768 patients and 113,383,368 controls. World Psychiatry.

[65] Bernardini F., Attademo L., Trezzi R., Gobbicchi C., Balducci P.M., Del Bello V., Menculini G., Pauselli L., Piselli M., Sciarma T. (2019). Air pollutants and daily number of admissions to psychiatric emergency services: Evidence for detrimental mental health effects of ozone. Epidemiol. Psychiatr. Sci..

